# Cryodebulking to Relieve Critical Airway Narrowing Due to a Central Airway Tumor at the Carina: A Case Report and Literature Review

**DOI:** 10.7759/cureus.53762

**Published:** 2024-02-07

**Authors:** Vineet Simhan, Srivatsa Lokeshwaran, Nitesh Gupta, Uzair Baig, Susmita Rakshit

**Affiliations:** 1 Pulmonology, Aster Hospital Whitefield, Bangalore, IND; 2 Interventional Pulmonologist, Aster Hospital Whitefield, Bangalore, IND; 3 Pulmonary, Critical Care, and Sleep Medicine, Vardhman Mahavir Medical College (VMMC) and Safdarjung Hospital, Delhi, IND; 4 Pathology, Aster Hospital Whitefield, Bangalore, IND

**Keywords:** rigid bronchoscopy, squamous cell lung carcinoma, central airway tumors, critical airway stenosis, cryodebulking

## Abstract

Central airway tumors presenting as critical airway stenosis is a medical emergency. Employing a cryoprobe, we successfully debulked a central airway tumor, providing rapid relief to a patient who came to the emergency room with severe breathlessness, hemoptysis, and respiratory failure. The current report underscores the efficacy of cryodebulking as an immediate and minimally invasive technique and a compelling alternative to conventional heat-based therapies.

## Introduction

Critical airway stenosis is an emergent condition that is often caused by central airway tumors. These may be due to metastasis from primary lung cancers, primary tracheobronchial tumors, or parenchymal tumors compressing the trachea and bronchus [1,2]. Nearly 40% of lung cancer patients undergo some form of central airway obstruction that may ultimately lead to critical respiratory failure or death. In most cases, the diameter of the trachea is already significantly reduced (<8 mm) before the development of symptoms, due to which up to 54% of patients present in respiratory distress [2]. Due to the late presentation, effective management is generally aimed at palliative care rather than with curative intent [3,4]. Patients presenting with respiratory failure due to critical airway stenosis have traditionally been treated with thermo-ablative techniques (electrocautery, laser, and argon plasma coagulation (APC)) followed by metallic or synthetic stent placement, with or without extracorporeal membrane oxygenation (ECMO) as a stop-gap until re-establishing a patent airway [3,5]. Our case underscores the efficacy of cryorecanalization as an immediate treatment in alleviating critical stenosis in a patient who presented with respiratory failure due to a large central airway tumor at the level of the carina. This approach also facilitated ventilation through the side port of the rigid bronchoscope while circumventing the need for ECMO.

## Case presentation

**Figure 1:**
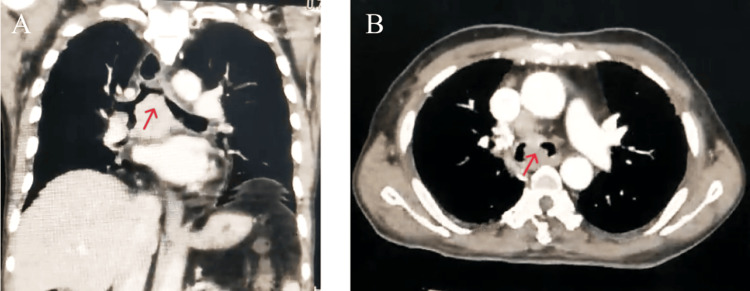
Computed tomography of the chest demonstrating a central airway tumor (arrow) at the level of the carina in the coronal view (A) and axial view (B)

A 78-year-old male with comorbidities of diabetes and hypertension, a current smoker (40-pack years), presented to the emergency department with progressive breathlessness, cough, hemoptysis, and weight loss over the last 20 days. He developed stridor over the last two days prior to presentation. On examination, the patient had stridor, tachypnea, and bilateral coarse crepitations on chest examination. Laboratory investigations were within normal limits. The computed tomography of the chest showed a large central airway mass at the level of the carina, infiltrating the right and left main bronchus, causing critical airway stenosis of both the mainstem bronchi (Left: 1 mm, Right: 1.2 mm) (Figures 1A, 1B). A multi-disciplinary discussion was conducted and agreement was reached on proceeding with rigid bronchoscopy for debulking the tumor for relief of airway obstruction and establishing a histopathological diagnosis.

**Figure 2:**
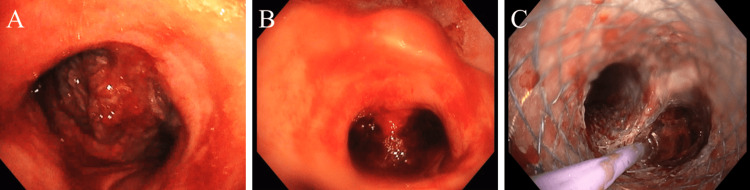
(A) Bronchoscopic view of a central airway tumor at the level of the carina obstructing visualization of the distal airway and right and left main bronchi. (B) View post cryodebulking of the tumor, restoring airway patency and allowing visualization of the left and right main bronchi. (C) Visualization of the metallic Y-stent at the level of the carina, post cryodebulking.

The patient underwent rigid bronchoscopic intubation using a 13 mm rigid tracheal barrel (Effer series 2). After ventilating through a rigid bronchoscope, a flexible bronchoscope, an Olympus adult BF-1TH 1100 bronchoscope (OD-6.1 mm, ID-5.8 mm, and WC-3.0 mm; Olympus Medical Systems, Tokyo, Japan) was deployed through a rigid bronchoscope barrel. The carinal tumor obstructed visualization of the distal airway (Figure 2A). The 1.7 mm cryoprobe was introduced through the working channel of the bronchoscope, and using 6 seconds freeze-thaw cycles, debulking of the tumor was done piecemeal. The tip or sides of the probe were approximated with the surface of the tumor which helped in rapid sequential debulking, thereby giving us enough flexibility at the site, in contrast to thermal ablative techniques. Subsequently, debulking of the left main bronchus tumor was done until a suitable airway lumen was reestablished and samples were sent for histopathological analysis. The process was repeated for the right main bronchus and the carinal obstruction, restoring airway patency and obtaining an acute angle at the carina for stent placement (Figure 2B). Airway dilatation was done following debulking with the help of a CRE^TM^ esophageal dilatation balloon 10-11-12 mm (3, 5, and 8 atm; Boston Scientific, Marlborough, Massachusetts, USA) so that adequate lumen patency was established, following which hemostasis was achieved. Clearance of secretions and blood was done through the bronchoscope.

The necessity for inserting an airway stent was discussed with the patient's family to prevent regrowth of the tumor, following which consent was given. A covered self-expanding metal stent (Mitra Industries, Faridabad, Haryana, India) - 40 mm (length) by 12 mm (diameter) by 16 mm (bronchial length) was placed under fluoroscopic vision. The flexible bronchoscope was introduced to check the position of the stent, and the airway distal to the landing space was visualized (Figure 2C). The rigid bronchoscope was then gradually withdrawn.

**Figure 3:**
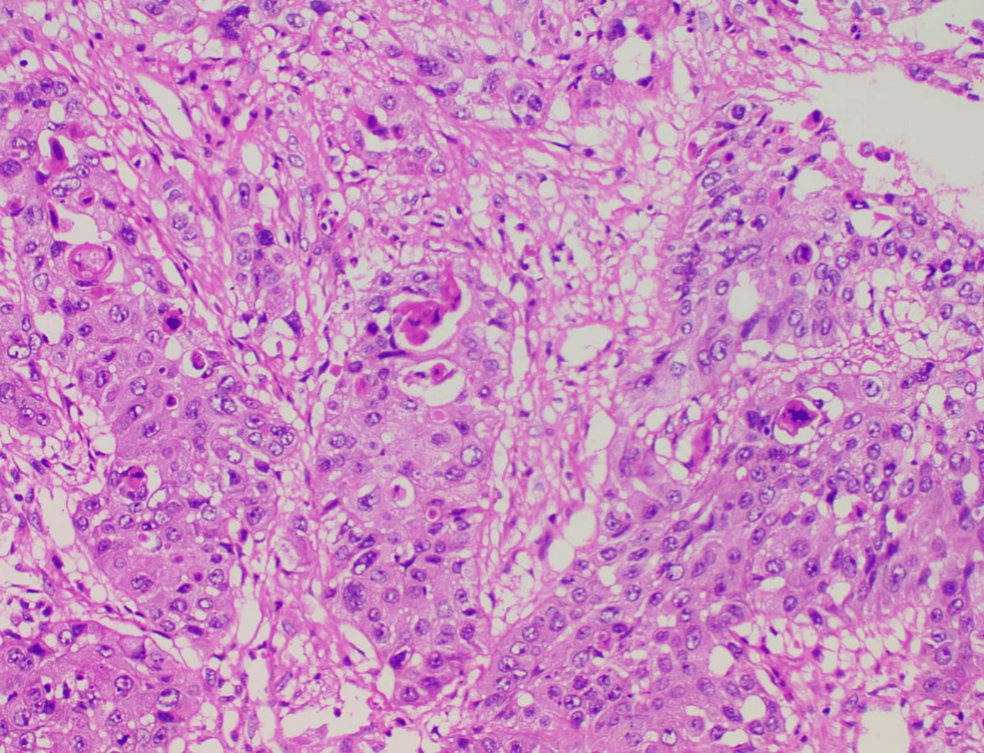
Tumour mass composed of round to polygonal epithelial cells arranged in solid sheets, trabeculae, and clusters of pleomorphic neoplastic cells with a large nucleus, dense eosinophilic cytoplasm, and atypical mitoses. Intercellular bridges seen. Desmoplastic reaction present. 20 x magnification; staining agent used - hematoxylin and eosin

Post-procedure, the general anesthesia was reversed and the patient was extubated on the table after noting good breathing mechanics. Post-extubation, we observed no stridor with significant improvement in his breathlessness. The patient was mobilized with the help of physiotherapy and there was significant improvement in activities of daily living. Histopathological analysis of the specimen obtained showed a moderately differentiated squamous cell carcinoma (Figure 3). Follow-up at three months with a CT scan and repeat bronchoscopy was status quo.

## Discussion

Patients facing an acute, critically threatened airway require immediate intervention and stabilization with a ventilating rigid bronchoscope [6]. Bronchoscopy helps in precise lesion localization and tissue sampling for histopathological analysis and allows access for various methods of tumor debulking.

Tumor debulking (through heat-based therapies or cryorecanalization) via a bronchoscope is the mainstay of treatment [7]. Cryorecanalization utilizes the Joules-Thompson effect, freezing the probe tip at -104^0^F to remove tumors en bloc [6]. This process is repeated until complete tumor removal and relief of airway obstruction [8]. In our case, cryodebulking was chosen over heat-based therapies due to the tumor's location and the need to minimize potential complications.

Cryotherapy's efficacy, linked to tissue water content, makes it a safer ablation method for the airway to prevent damage to cartilage-like tissues, reducing the risk of complications such as airway perforation compared to traditional heat-based techniques [7]. Heat-based therapies require a low fraction of inspired oxygen (FiO_2_) (< 0.4) to prevent airway fires. These become contraindicated when elevated FiO_2_ levels are needed to provide adequate ventilation [9]. Cryorecanalization was hence chosen as a safer method for alleviating the stenosis in our case while allowing the maintenance of elevated FiO_2_ levels during the debulking process. Cryodebulking also offers the added advantage of preserving tissue samples without causing destruction, which facilitates a more accurate histopathological analysis such as in our case where a biopsy was needed [10]. Either the tip or side of the cryoprobe can be approximated with the tumor, leading to increased flexibility at the site of the tumor, resulting in more efficient debulking of the tumor. This cannot be achieved with traditional electrocautery or a snare. A comparison between cryodebulking and heat-based therapies is summarized in Table 1.

**Table 1:** Comparison between cryodebulking and heat-based therapies FiO_2_: fraction of inspired oxygen

Cryodebulking	Heat-based therapies
Advantages	Advantages
Less risk of airway perforation	Less risk of bleeding (heat aids in hemostasis)
Preserves tissue sample	Central suction can clear blood and debris from the airway
Ability to maintain sufficient FiO2	
Reduced rate of airway fires and electrical accidents	
Disadvantages	Disadvantages
Intrabronchial bleeding	Perforation risk
Pneumothorax risk	Need to keep FiO_2_ < 40% to prevent airway fires or electrical accidents
Bronchospasm	Coagulation of tissue can result in subpar samples for histopathological analysis
Arrhythmia risk	Reduced flexibility of probe

The predominant adverse effect of cryorecanalization is intrabronchial bleeding, with most cases being mild and self-resolving. In instances of more severe hemorrhage, effective management can be easily achieved through the use of argon plasma coagulation (APC) [11,12]. Table 2 summarizes the incidence of bleeding in three different studies where cryorecanalization was carried out. Backup options with heat-based methods were available to us if cryodebulking resulted in significant bleeding or complications.

**Table 2:** Comparison of bleeding in patients undergoing cryodebulking APC: argon plasma coagulation

Name of study	No. of patients undergoing cryorecanalization	% of successful interventions (patients)	% of mild bleeding after intervention (patients)	% of moderate bleeding after intervention requiring APC (patients)
Hetzel et al. [9]	60	83% (50)	90% (54)	10% (6)
Aydın Yilmaz et al. [11]	40	72.5% (29)	37.5% (15)	25% (10)
Jong Hwan Jeong et al. [12]	233	90% (211)	5% (12)	27.9% (65)

## Conclusions

Critical airway obstruction arising from central airway malignancies constitutes a medical emergency, demanding swift diagnosis and intervention. Endobronchial therapies, particularly cryodebulking, emerge as pivotal for substantial relief, showcasing high success rates and minimal complications. In our case, a patient with critical central airway narrowing was successfully managed through cryorecanalization, followed by Y-metallic stent insertion. This choice was influenced by factors such as tumor location, size, safety profile, and the imperative to preserve tissue for biopsy. The intervention promptly restored airway patency, alleviating breathlessness and hemoptysis symptoms with minimal adverse effects, ultimately leading to an improved quality of life in follow-up appointments. The success of this approach underscores its efficacy in addressing critical airway obstructions.
